# Breast carcinoma in a dog: sensitivity and specificity between cytopathology and histopathology

**DOI:** 10.29374/2527-2179.bjvm003024

**Published:** 2024-09-18

**Authors:** Paola Alejandra Montenegro Cuellar, Noeme Sousa Rocha, Natália Freitas de Souza, Fernando Carmona Dinau

**Affiliations:** 1 Veterinarian, Programa de Pós-graduação em Patologia Veterinária, Departamento de Clínica Veterinária, Faculdade de Medicina Veterinária e Zootecnia, Universidade Estadual Paulista (UNESP), Botucatu, SP, Brazil.; 2 Veterinarian, DSc., Programa de Pós-graduação em Patologia Veterinária, Departamento de Clínica Veterinária, Faculdade de Medicina Veterinária e Zootecnia, UNESP, Botucatu, SP, Brazil.; 3 Veterinarian, MSc., Programa de Pós-graduação em Patologia Veterinária, Departamento de Clínica Veterinária, Faculdade de Medicina Veterinária e Zootecnia, UNESP, Botucatu, SP, Brazil.; 4 Undergraduate in Veterinary Medicine, Faculdade de Medicina Veterinária e Zootecnia, UNESP, Botucatu, SP, Brazil.

**Keywords:** carcinoma, cell, cytopathology, female dog, mamma, carcinoma, célula, citopatologia, cadela, mama

## Abstract

This study evaluated the accuracy of mammary carcinoma diagnoses in female dogs through cytological exams (FNA) compared to histopathological diagnoses. The presence of neoplasia and the effectiveness of procedures at the Pathology Laboratory of the Veterinary Hospital of the FMVZ of Unesp Botucatu, were analyzed. Between 2015 and 2020, a total of 1100 mammary neoplasms were identified, of which 569 were mammary carcinomas. Fifty cytological samples were selected and analyzed to determine occurrence, age at presentation, and the most affected breeds, as well as to verify the obtained diagnoses. Mammary carcinoma constituted for 51.72% of the registered cases. A higher occurrence was observed in mixed-breed female dogs, at 40.42%, followed by Poodles at 17%. The most common age at diagnosis was 10 years, and in 65.55% of cases, the dogs had not been previously spayed. 9.31% of the animals had received contraceptives, while 14% had given birth and 14.58% had presented symptoms of pseudopregnancy at some point in their lives. In the test results, a 70% agreement between cytology and histology was observed, with a 30% disagreement between them. Statistically, a sensitivity of 79.32% and a specificity of 57.14% were reflected. Intact and older female dogs represent a significant risk of developing mammary carcinoma. Although the protocol for processing and interpreting cytological samples is well established, the results do not reach the level of excellence observed in previous studies.

## Resumo

O estudo avaliou a precisão dos diagnósticos de carcinoma mamário em cadelas por meio de exames citológicos (PAAF) em comparação com diagnósticos histopatológicos. Foi analisada a presencia da neoplasia e a eficácia dos procedimentos no Laboratório de Patologia do Hospital Veterinário da FMVZ da Unesp, Botucatu. Foram registradas 1100 neoplasias mamárias entre 2015 e 2020, das quais 569 foram carcinomas mamários. Cinquenta amostras citopatológicas foram selecionadas e analisadas para determinar a ocorrência, idade de apresentação e raças mais afetadas, além de verificar os diagnósticos obtidos. O carcinoma mamário representou 51,72% dos casos registrados. Observou-se maior ocorrência em cadelas SRD, com 40,42%, seguidas por Poodles com 17%. A idade mais comum de diagnóstico foi de 10 anos, e em 65,55% dos casos as cadelas não haviam sido castradas previamente. 9,31% dos animais haviam recebido contraceptivos, enquanto 14% já haviam dado à luz e 14,58% apresentaram sintomas de pseudociese em algum momento de suas vidas. Nos resultados dos exames, foi observada uma concordância de 70% entre citologia e histologia, com 30% de discordância entre eles. Estatisticamente, foi refletida uma sensibilidade de 79,32% e uma especificidade de 57,14%. Cadelas intactas e mais velhas representam um risco significativo no desenvolvimento de carcinoma mamário. Embora o protocolo de processamento e interpretação de amostras citopatológicas esteja bem estabelecido, os resultados não alcançam o nível de excelência observado em estudos anteriores.

## Introduction

Cancer arises from an imbalance in the process of cell reproduction, resulting in uncontrolled cell proliferation. These cells form neoplasms, which can be benign or malignant (causing cancer), potentially disrupting disrupt bodily functions. Among the various types of cancer, those originating from epithelial cells of organs are the most common, with breast cancer being predominant (American Cancer Society, 2020).

Breast cancer is one of the most diagnosed cancers in women worldwide, as well as in female dogs, being malignant in 50% of cases and having a morbidity rate three times higher compared to women (Egenvall et al., 2005; Jadotte et al., 2021; Siegel et al., 2017).

Most data indicate that early spaying (ovariohysterectomy, OHE) in female dogs reduces the risk of developing mammary tumors. Furthermore, if an intact animal develops mammary tumors, performing spaying simultaneously with mastectomy significantly increases the animal's survival compared to spaying alone (Banchi et al., 2022; Stavisky & White, 2022).

Understanding diagnostic methods and performing a thorough physical examination are essential for enhancing the animal's quality of life. These practices help alleviate suffering and, depending on the disease's progression, can sometimes lead to a cure (Santos, et al. 2023).

Fine needle aspiration (FNA) has several advantages, including high sensitivity and specificity, low morbidity, rapid diagnosis, and a favorable cost-benefit ratio. However, its main disadvantages include the lack of experience in cytological interpretation and the absence of a standardized and uniform system for reporting FNA results (Domansk, 2020).

This study aimed to determine the sensitivity and specificity of cytopathological and histopathological diagnoses of breast cancer in female dogs by analyzing 50 cases, reevaluating the diagnoses according to literature criteria, comparing them with an updated bibliography, and determining the frequency of breast carcinoma in female dogs.

## Material and methods

The research project was approved by the Ethics Committee for Animal Use of the FMVZ at UNESP, Botucatu Campus, under protocol number 0190/2022 - CEUA.

Through a retrospective longitudinal descriptive study, records from the Veterinary Pathology Laboratory of the FMVZ Veterinary Hospital (VH) were analyzed to select and review all cases of mammary neoplasia in female dogs from annual requisitions between 2015 and 2020.

The inclusion criteria for determining the frequency and presentation of breast carcinoma included detailed clinical data such as breed, age, reproductive history, and disease progression anamnesis.

From these cases, 50 were selected to represent the cytological diagnosis, along with the corresponding histopathological examinations, allowing for microscopic evaluation and comparison to determine the sensitivity and specificity of cytology. The cytological analysis was performed according to the criteria adopted by Raskin et al. (2022) to compare its equivalence with diagnoses based on the standardized classification of Cassali et al. (2020) for histopathological diagnosis.

Case data were recorded using software tools such as Excel to facilitate comparison between cytological and histopathological diagnoses. This enabled the determination of the percentage of agreement between the nomenclatures and the analysis of the sensitivity and specificity of cytology compared to histopathology.

Cytological and histological samples were analyzed using the Opticam® O400S image analyzer with Mosaic 2.4 software at 40x magnification. Finally, the effectiveness of the cytological criteria suggested in the literature (Raskin et al., 2022) for identifying malignant mammary neoplasms in female dogs was evaluated.

The methods described by Trevethan (2017) were used to analyze the data and calculate Sensitivity, Specificity, Positive Predictive Value (PPV) and Negative Predictive Value (NPV). Sensitivity was defined as the probability of correctly diagnosing a cancer case, while specificity was the probability of correctly diagnosing a non-cancer case.

PPV represents the probability that suspected cancer cases are indeed cancer, while NPV indicates the probability that suspected non-cancer cases are truly not cancer.

## Results

**Figure 1:**
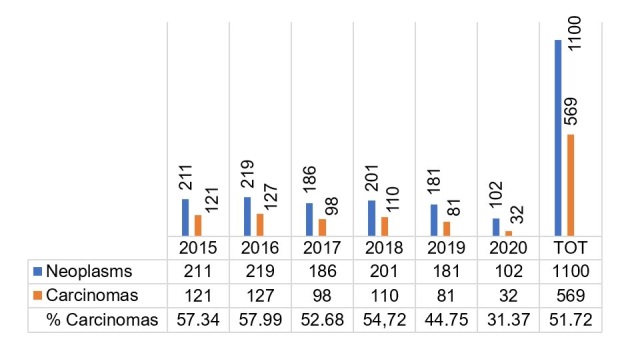
Total neoplasms and carcinomas.

*Note*. Number of cases of annual neoplasms and breast carcinomas with their respective totals and percentages.

Between January 2015 and December 2020, 7,430 female dogs were examined. Of these, 1,100 cases had neoplastic lesions in the mammary glands (14.80%), with 569 cases diagnosed as mammary carcinomas (51.72%) (Figure 1).

**Figure 2:**
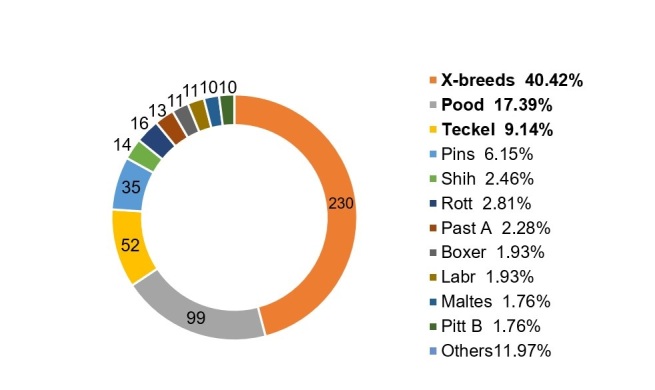
Most diagnosed breeds.

*Note*. Presenting 11 of the 37 breeds most diagnosed with mammary carcinoma, the X-breed leads with 40.42%, followed by the Poodle with 17.39% and Dachshund with 9.14%.

**Figure 3:**
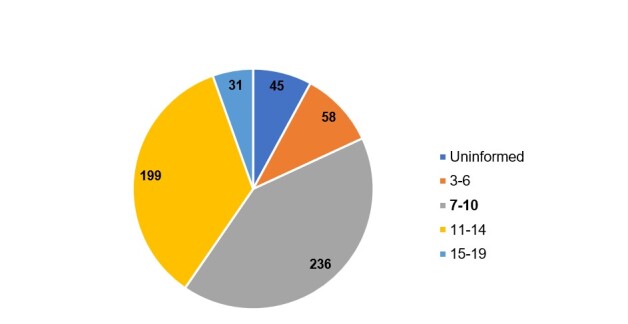
Age Range.

*Note*. The identities of patients diagnosed with mammary carcinoma are highlighted, as are the age ranges with the highest incidence of cases. Uninformed: the owners do not know the identity of their pet.

Among the 569 diagnoses of mammary carcinoma, mixed-breed dogs led with 230 cases (40.42%), followed by Poodles with 99 cases (17.39%), and Dachshunds with 52 cases (9.14%) (Figure 2). The age group with the highest number of carcinoma cases was 7 to 10 years, with a total of 236 cases, followed by 11 to 14 years with 199 cases (Figure 3). Thus, the age with the most carcinoma diagnoses was 10 years, with 77 patients (13.53%), followed by 11 years (13%), 9 years (11.07%), and 8 years (8.78%).

Despite the diagnosis, 188 patients were not subjected to OHE (33.04%), while 185 patients underwent the procedure after diagnosis (32.51%). Together, 373 patients had not had OHE before diagnosis, representing 65.55% of the total. Additionally, 88 patients, whose owners were unaware of their reproductive history due to being adopted adult animals, accounted for 15.46% of the total.

According to reports from caretakers, 53 patients received contraceptives (9.31%). However, information on the duration or type of contraceptive used was not available. Furthermore, a total of 80 patients had litters (14.05%), while 83 experienced pseudocyesis (14.58%) (Table 1).

**Table 1:** Information on the patient’s reproductive history.

**Reproductive History**	**No. of Patients**	**%**
**No OHE until Diagnosis**	**373**	**65.55**
OHE Before Diagnosis	108	18.98
Owner's lack of knowledge	88	15.46
Use of Contraceptives	53	9.31
Puppies	80	14.05
Pseudocyesis	83	14.58

*Note*. Low rate of castration prior to diagnosis, in addition, a significant percentage did not undergo castration even after diagnosis.

After reviewing 50 cytological and 50 histopathological samples from the patients, initially, 23 cytologies suggested the presence of cancer, which was confirmed by histopathology. Moreover, 9 cytological samples suspected of cancer revealed other types of neoplasms, such as myoepitheliomas, mast cell tumors, hemangiosarcomas, and hemangiomas.

**Figure 4:**
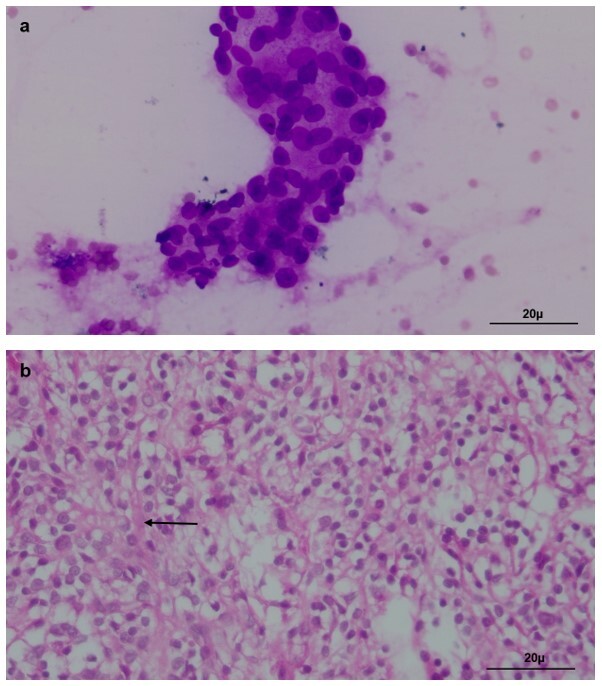
Solid Carcinoma: (a) Cohesive epithelial cells with minimal cellular atypia, emphasizing the importance of histopathological confirmation in suspected neoplasia cases (H&E 40x); (b) Proliferation of epithelial cells in a solid arrangement, bordered by fibrovascular connective tissue (arrow) (H&E 40x).

*Note.* Solid Carcinoma: (a) Cohesive epithelial cells with minimal cellular atypia, highlighting the importance of histopathological confirmation in suspected neoplasia cases (H&E 40x); (b) Proliferation of epithelial cells in a solid arrangement, bordered by fibrovascular connective tissue (arrow) (H&E 40x).

**Figure 5:**
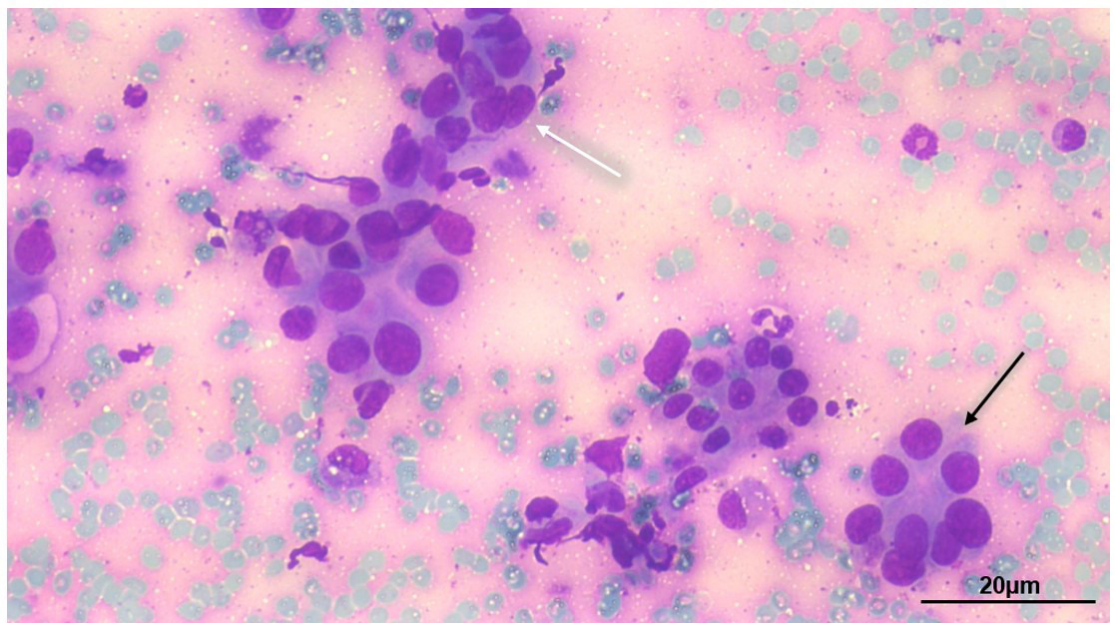
Cells arranged in cohesive groups, showing cellular pleomorphism, scarce and poorly defined cytoplasm, an increased nucleus/cytoplasm ratio, anisocytosis, and macrocytosis; nuclear molding (white arrow), acinar arrangement (black arrow), (H&E 40x).

*Note*. Cells arranged in cohesive groups, cellular pleomorphism, scarce and poorly defined cytoplasm, increased nucleus/cytoplasm ratio, anisocytosis and macrocytosis; nuclear molding (white arrow), acinar arrangement (black arrow), (H-E 40x).

**Figure 6:**
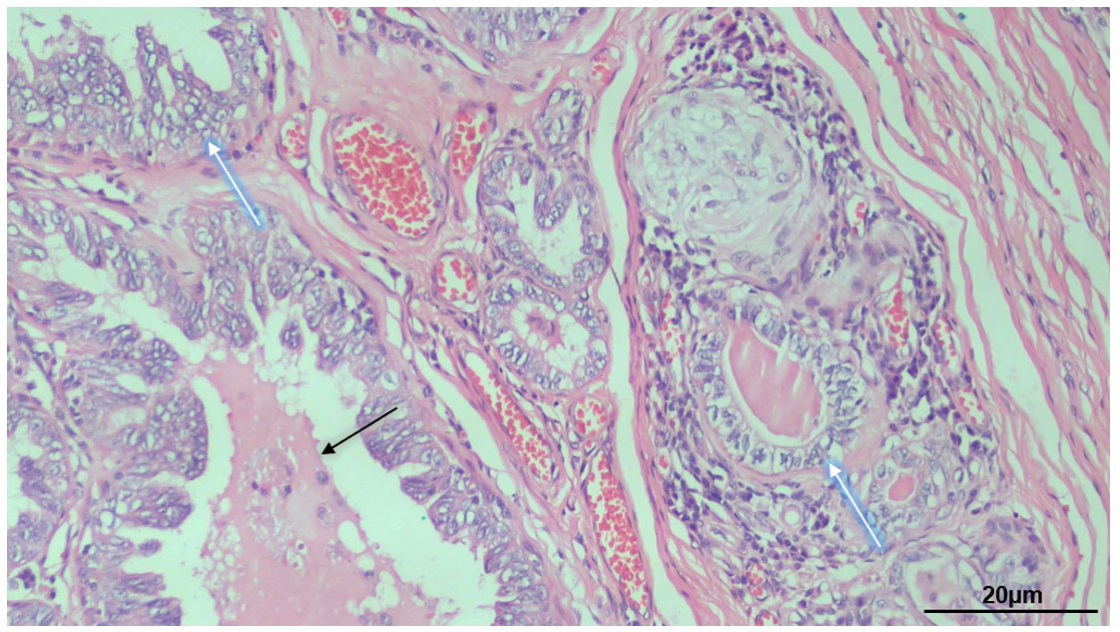
Cubic to columnar epithelial foci with high pleomorphism (white arrows) and cartilaginous and bone components (black arrow), in addition to changes in the nucleus-cytoplasm ratio in mixed tumor carcinoma (H&E 40x).

*Note.* Cubic to columnar epithelial foci with high pleomorphism (white arrows) and cartilaginous and bone components (black arrow), in addition to changes in the nucleus-cytoplasm ratio in mixed tumor carcinoma (H-E 40x).

**Figure 7:**
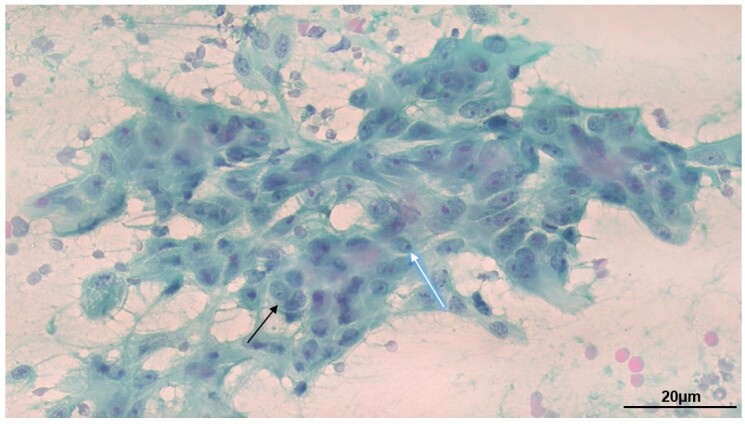
Multinucleated cell (black arrow), prominent nucleolus (white arrow) (Pap 40x).

*Note.* Multinucleated cell (black arrow), evident nucleolus (white arrow) (Pap 40x).

Representative images of the cases used during the analysis of cytological and histological samples show some malignancy criteria used for the diagnosis of breast carcinoma (Figures 4, 5, 6 and 7). In Figure 4, cytologically (a) an increase in the size and number of mammary glandular cells was observed, with preserved morphology and no significant atypical alteration, suggesting mammary hyperplasia.

Histologically (b), an increase in the number and size of mammary glandular structures was found, along with malignant cells invading the stroma and forming solid or trabecular structures with irregular borders, abnormal vascularization, and some invasion of blood and lymphatic vessels, confirming a solid breast carcinoma.

On the other hand, in 12 cytological samples where other types of neoplasms were suspected, confirmation was achieved through the corresponding histological analysis. There was disagreement with the initial cytological diagnosis in only 6 histopathological samples.

In total, there were 35 agreements (70%) between the cytological and histopathological examinations, whereas there were 15 disagreements (30%). Therefore, the samples sensitivity was 79.31%, and the specificity was 57.14%. Additionally, the positive predictive value (PPV) was 71.87%, while the negative predictive value (NPV) was 66.66% (Table 2).

**Table 2:** Sensitivity, specificity and positive and negative predictive values.

			Histopathology		
		a	b	Total	
		True Positives: 23	False Positives: 9	32	
Cytopathology		c	d		
		False Negatives: 6	True Negatives: 12	18	
		Total			
		29	21	50	
				
Sensitivity:	23	x 100 = **79.31%**	PPV:	23	x 100 = **71.87%**
	23+6			23+9	
					
Specificity:	12	x 100 = **57.14%**	NPV:	12	x 100 = **66.66%**
	9+12			6+12	

*Note*. Histopathology (“Gold Standard”) is the reference exam that tells us whether cytopathological diagnoses are correct.

In this study, precise data on lesion location could not be obtained. Records for 349 examined dogs lacked adequate descriptions of the precise lesion locations, making it difficult to identify the most affected mammary glands. In some cases, descriptions were vague, using generic terms such as “mammary chain” (14.58%) and “mammary lesion” (24.06%).

## Discussion

This study highlights that breast carcinoma in female dogs represented a significant proportion, with 51.72% of cases, according to Vazquez et al. (2023), who observed an occurrence between 50% and 70% in unneutered dogs. Although the total number of neoplasia cases represents less than a quarter of the total consultations at the Veterinary Hospital annually, breast carcinomas constitute more than half of the neoplasia cases treated.

Moreover, a significant proportion of the sample consisted of mixed-breed dogs, namely 40.42%, corroborating similar findings in previous studies (Carvalho et al., 2023; Edmunds et al., 2023; Oliveira et al., 2022). Mixed breeds consistently showed the highest occurrence, probably due to their predominance in the canine population, followed by Poodles.

In female dogs, mammary neoplasia typically occurs around 9 years of age, which is equivalent to 60 to 90 years in humans (Sultan & Ganaie, 2018). According to Edmunds et al. (2023), previous studies have indicated that breast carcinoma in female dogs is more prevalent at the age of 10 years, with an average age of 7 to 10 years. This study supports this trend as the majority of affected dogs are aged between 7 and 14 years, with an average age of approximately 10 years.

In 99.47% of cases, female dogs with mammary tumors were not spayed or were not spayed before their first heat. This finding aligns with previous studies, highlighting a significant proportion of patients who do not undergo spaying (Nunes et al., 2018; Oliveira et al., 2022). These results are in contrast to studies conducted in the United Kingdom, where a smaller proportion of females remain intact (Edmunds et al., 2023).

Among the 50 cases selected for cytopathological and histopathological evaluation, some cases exhibited multiple histological neoplasms. In these cases, mammary hyperplasia was observed alongside solid carcinoma and mast cell tumors in association with hemangioma within the same region.

This finding is consistent with recent studies that have documented concurrent malignant and benign neoplasms, as well as the coexistence of two or more distinct histological subtypes (11.5%) (Dolka et al., 2018).

The present study demonstrates the efficacy of fine needle aspiration (FNA) cytology in detecting truly present mammary lesions. However, its specificity of 57.14% suggests the possibility of some false positives. The positive predictive value (PPV) of 71.87% is encouraging, indicating that most lesions diagnosed as positive by FNA are true positives, while the negative predictive value (NPV) of 66.66% suggests the presence of false negatives.

In comparison to previous research (Chen et al., 2022), FNA proved to be a reliable technique for diagnosing palpable mammary lesions, achieving 100% specificity and PPV, 93.3% sensitivity, and 98.2% NPV. FNA can provide accurate diagnoses for most soft tissue neoplasms, especially with rigorous cytological criteria, adequate samples, and access to auxiliary techniques and clinical and radiological data (Domansk, 2020).

The divergent results between the two studies indicate a significant difference, possibly due to variations in methodology, from sample collection and processing to data interpretation. This suggests a possible need to improve the existing laboratory protocol.

Besides specific patient history details and comprehensive clinical examinations of the mammary glands, a solid theoretical understanding of mammary tumors in female dogs is essential. It is also crucial for veterinarians to receive practical training in collecting adequate samples, including precise puncture techniques, identification of tumor quadrants, and proper use of necessary materials.

Recently, the effectiveness of the Yokohama System has been demonstrated by the International Academy of Cytology in classifying fine needle aspiration biopsies of mammary lesions. (Nikas et al., 2023). This study emphasizes the importance of rapid in situ evaluation whenever possible, reducing inaccurate interpretations and maximizing the identification of benign and malignant lesions, presenting a model that could be adopted.

## Conclusion

The findings emphasize that non-spayed female dogs have a high incidence of cancer, highlighting age as an important risk factor for tumor development. Additionally, it is crucial to consider other risk factors, such as the use of contraceptives and the occurrence of pseudocyesis, which may not have been fully examined in previous studies.

The low sensitivity and specificity values indicate significant deficiencies in the diagnostic procedures' ability to accurately detect both positive and negative cases of mammary carcinoma in female dogs.
